# Glutathione sulfotransferase inhibition activity of a self-fermented beverage, *Kanji*

**DOI:** 10.1080/13880209.2016.1257030

**Published:** 2016-12-12

**Authors:** Abida Latif, Khalid Hussain, Naureen Shehzadi, Muhammad Islam, Muhammad Tanveer Khan, Rukhsana Anwar, Humaira Majeed Khan, Nadeem Irfan Bukhari

**Affiliations:** a Faculty of Pharmacy, University of the Punjab, Lahore, Pakistan;; b School of Pharmaceutical Sciences, Universiti Sains Malaysia, Penang, Malaysia;; c Institute of Pharmacy, Lahore College for Women University, Lahore, Pakistan

**Keywords:** Ferulic acid, HPLC, pharmacokinetics, *Daucus carota L*., apiaceae, characterization, standardization

## Abstract

**Context:***Kanji,* a liquid preparation of roots of *Daucus carota* L. ssp. *sativus* (Hoffm.) Arcang. var. *vavilovii* Mazk. (Apiaceae), may inhibit glutathione sulfotransferase (GST) activity due to ferulic acid content.

**Objectives:** GST inhibition activity and characterization of *Kanji* and methanol extract of *D. carota* roots, and oral absorption pattern of ferulic acid from *Kanji* in rats.

**Materials and methods:** GST inhibition activity of *Kanji* and methanol extract of *D. carota* roots in concentration range 0.001–100.00 mg/mL was determined using Sprague Dawley rat liver cytosolic fraction. Methanol extract upon column chromatography gave ferulic acid, which was used to characterize *Kanji* and determine its oral absorption pattern in Wistar rats.

**Results:** The GST inhibition activity of *Kanji* (100.00 μg/mL), methanol extract of *D. carota* roots (100.00 μg/mL) and tannic acid (10.00 μg/mL, positive control) was found to be 0.162 ± 0.016, 0.106 ± 0.013 and 0.073 ± 0.004 μM/min/mg, respectively. Different *Kanji* samples and methanol extract contained ferulic acid (0.222–0.316 mg/g) and 0.77 mg/g, respectively. Ferulic acid did not appear in plasma after oral administration of *Kanji.*

**Discussion:***Kanji* having solid contents 80.0 μg/mL, equivalent to 0.0025 μg/mL ferulic acid, does not inhibit the activity of GST. The oral administration of *Kanji,* in human equivalent dose (528 mg/kg, 16.67 μg ferulic acid), to rats indicated poor absorption of ferulic acid.

**Conclusion:***Kanji* having solid contents 14–36 mg/mL does not inhibit GST activity, hence may not interfere with drugs that are the substrates of GST, if taken concomitantly.

## Introduction

*Kanji,* a self-fermented beverage, is used as a traditional medicine in several Asian countries to treat gastrointestinal and liver disorders, and increase appetite (Baloch 1994). This remedy is prepared in the beginning of the summer season in homes for personal use and small-scale industry to be sold commercially by road side sellers. Its main ingredient, fresh roots of *D. carota* L. ssp. *sativus* (Hoffm.) Arcang. var. *vavilovii* Mazk. (Apiaceae) contains ferulic acid which is a known GST inhibitor (Das et al. 1984; Martono 2005). Therefore, the use of this beverage is expected to affect the efficacy of concomitantly used drugs, which are the substrates of GST.

Biological toxins, medicinal agents and xenobiotics are metabolized by phase I and II biotransformation reactions (Bown 1995; Lu 1996; Gibson & Skett 2001; Asha & Vidyavathi 2010). The concomitant use of agents, which share these enzymes, may affect each others efficacy/toxicity. Such probabilities are increased due to poly-pharmacy; the risk is 6% on consuming two products together and 50% upon taking five drugs at once (Asha & Vidyavathi 2010). The *Kanji* has long been consumed by both healthy and sick population as a refreshing drink and complementary medicine. Despite, such use, the literature is devoid of any report describing its GST inhibition activity. Therefore, it is imperative to investigate *Kanji* for GST inhibition activity, ferulic acid contents and oral absorption pattern of ferulic acid. These studies are expected to provide evidence to the volume (dose) of *Kanji* that can be taken without GST inhibition.

Due to a number of traditional uses (Weiner 1980; Chevallier 1996; Gazzani et al. 1998), pharmacological activities (Bishayee et al. 1995; Cao et al. 1996; Balasubramaniam et al. 1998; Sun et al. 2009; Latif et al. 2013; Bystrická et al. 2015; Chandra et al. 2015) and as an ingredient of *Kanji,* the plant has a great commercial significance that can further be enhanced, if the beverage is made available round the year. We have also investigated different types of *Kanji* samples for a number of analytical and antioxidant activities and found variation of chemical constituents and mass/mL (total solid, 14–38 mg/mL) among the samples, and instability (Latif et al. 2013). Obviously, such samples are expected to contain different amount of ferulic acid, hence exhibit different GST inhibition activity. To confirm this, there is a dire need of finding ferulic acid contents of *Kanji* that can lead to determine the volume of the beverage which can be taken without GST inhibition. On the other hand, ferulic acid is reported to be poorly absorbed from the diet containing its lower amount (Adam et al. 2002). However, studies conducted on pure ferulic acid indicated fast absorption from different parts of the gastrointestinal tract (Spencer et al. 1999; Zhao et al. 2004). Therefore, it may further be useful to investigate how much of ferulic acid can be absorbed after oral administration of *Kanji*.

## Materials and methods

### Plant material

Black carrots were purchased from the local vegetable market in March, 2013 and authenticated by Prof. Dr Zaheer ud Din Khan, Department of Botany, Government College University, Lahore, Pakistan. A voucher specimen was deposited in Department of Botany, Government College University, Lahore, Pakistan, vide reference No. G. C. Bot. Herb. 958. The carrots were rinsed in water to remove extraneous matter and the residual water was dried using an electric fan. One part of these carrots was used to prepare *Kanji* and other was crushed and dried for extraction.

### Chemicals and solvents

Analytical/HPLC grade chemicals and solvents used were methanol, acetonitrile, formic acid, sodium dihydrogen phosphate, phosphoric acid, petroleum ether, chloroform, ethyl acetate, butanol, hexane, acetone, 1-chloro-2, 4-dinitrobenzene, reduced glutathione (GSH), bovine serum albumin, silica gel and silica gel 60F_254_ TLC plates (Sigma Aldrich and Merck, Darmstadt, Germany). Other materials of Merck included potassium dihydrogen orthophosphate, di-potassium hydrogen phosphate, tannic acid, calcium chloride, diethyl ether, barium hydroxide, potassium chloride, potassium hydroxide, sodium carbonate, sodium hydroxide, copper sulphate, potassium sodium tartrate, formalin and Folin–Ciocalteau’s reagent.

### Preparation of *Kanji* and collection of samples

The beverage termed as a Lab-made *Kanji* was prepared by a method described earlier (Latif et al. 2013). Briefly, 113 g vertically sliced thin-long pieces of fresh roots and 5 g each of red chillies, mustard seeds and table salt (sodium chloride) were added in a glass jar containing 1.50 L water. The jar was covered and the contents were allowed to ferment spontaneously at room temperature for 4 days (Berry et al. 1989; Sahota et al. 2008). Six samples of the beverage were collected from roadside sellers, whereas a homemade sample was gifted by a resident of Lahore, Pakistan. All the samples were stored at 10–15 °C until used.

### Extraction and fractionation

The dried root material (800 g) was extracted sequentially with petroleum ether, chloroform and methanol using the Soxhlet apparatus. The extracts were filtered and dried *in vacuo* at 40 °C. Methanol extract was fractionated by partitioning using solvents in the order of ascending polarity. Hexane, chloroform, ethyl acetate and butanol fractions were dried *in vacuo* at 40 °C, whereas water fraction was dried in a freeze dryer.

### Assessment of GST inhibition activity

#### Rat-liver cytosolic fraction and its protein contents

The study was performed according to the approved protocol of the Animal Ethics Committee, Universiti Sains Malaysia, Penang, Malaysia. Sprague Dawley rat’s liver obtained under diethyl ether anaesthesia was rinsed successively in ice-cold water and ice-cold potassium phosphate buffer (pH 7.4), blotted, weighed and homogenized in a mixture of 67 mM potassium phosphate buffer (pH 7.4) and 1.15% potassium chloride solution (3:1, v/v), using a Potter-Elvehjem homogenizer. The homogenate was centrifuged at 12,500 *g* for 20 min at 4 °C using Optima TM TLX refrigerated ultracentrifuge (Beckman Coulter, Inc., Brea, CA). The supernatant was further centrifuged at 100,000 *g* for 60 min at 4 °C, and the supernatant obtained was the cytosolic fraction of the liver. The protein concentration of the cytosolic fraction was determined by a method described by Lowry et al. (1951).

#### Enzyme activity assay

The enzyme activity was determined using a spectrophotometric method as described by Habig et al. (1974). Briefly, a reaction mixture containing 2.5 mL of 50 mM potassium phosphate buffer (pH 6.5), 0.2 mL of 20 mM reduced glutathione (GSH) and 0.15 mL of cytosolic fraction (protein concentration 0.125 mg/mL) was incubated with 0.1 mL aqueous solution of *Kanji/*methanol extract in a concentration range 0.001–100 μg/mL for 10 min at room temperature. The reaction was initiated by adding 0.15 mL of 1 mM 1-chloro-2,4-dinitrobenzene aqueous solution and the rate of thioester formation was determined by measuring the absorbance after 2 min at 340 nm. The reaction mixtures containing 0.1 mL tannic acid (10 μg/mL) and 0.1 mL water were served as a positive control and control sample, respectively. A blank was prepared such that the sample using 0.15 mL of water instead of cytosolic fraction. The samples, positive control and control were analyzed in triplicate and enzyme activity was calculated using an extinction coefficient (0.0096 μM^−1^ × cm^−1^) of the thioester formed by the enzyme.

#### Isolation and identification of ferulic acid

The silica gel (142 g), 40–60 μm mesh, activated in an oven at 110 °C for 1 h, was packed in a column (64 cm long, 2.5 cm outer diameter, 2.1 cm inner diameter) by the wet method using methanol. Hexane fraction of methanol extract (25 g), adsorbed on 5 g silica gel was loaded on the column. The elution was carried out with 900 mL mixture of methanol and ethyl acetate (70:30, v/v) at a flow rate of 30 drops/min, resulting in 18 fractions (A1–18), 50 mL each. Based on TLC profile, solvent system methanol and ethyl acetate (50:50, v/v), fractions A3–A7 were pooled, concentrated at 40 °C *in vacuo*, and subjected to pTLC, using mobile phase comprising methanol and ethyl acetate (50:50, v/v), which gave a compound (25 mg). The compound was investigated for purity by TLC and HPLC, which was then, identified using spectral and chromatographic data.

### Determination of ferulic acid by HPLC

#### Preparation of solutions

A standard solution of ferulic acid (1.0 mg/mL) was prepared in HPLC grade methanol, which was then diluted with methanol to produce a series of standard solution (10.0, 20.0, 40.0, 60.0, 80.0 and 100.0 μg/mL).

Sample solutions of freeze-dried *Kanji*, extracts and fractions (5 mg/mL) were prepared in HPLC grade methanol. The sample/standard solutions were filtered using 0.45 μm syringe filters (Whatman, Maidstone, England).

#### HPLC analysis

HPLC system: 1200 series, equipped with isocratic pump (G1310 A), auto sampler (G1329 A), column oven (G1316 A), DAD (G 1315 B) and fluorescent light detector (G1321 A) of Agilent Technologies, Waldron, Germany, was used in the present study. Each standard/sample solution (20 μL) was eluted at a flow rate of 1.0 mL/min through a column – Eclipse X DB-C_18_ (5 μm, 4.6 × 150 mm) – using an isocratic mobile phase comprising water, methanol and phosphoric acid (300:200:2.5, v/v/v). The temperature of the column was maintained at 25 °C and detection was carried out using FLD; operated at 250 nm excitation and 410 nm emission. Data acquisition was performed with LC/MS ChemStation.

#### Oral bioavailability of ferulic acid from Kanji

##### Animals

Six male Wistar rats, weighing 280 ± 20 g, were obtained from and kept in the Animal House, Punjab University College of Pharmacy, University of the Punjab, Lahore, Pakistan. Standard pellet diet and tap water was supplied *ad libitum*. The study protocol was duly approved by the Animal Ethics Committee, Punjab University College of Pharmacy, University of the Punjab, Lahore, Pakistan.

##### Dose preparation and administration.

Freeze-dried *Kanji* was dissolved in a mixture of water and PEG 400 (1:1, v/v) to get a solution of concentration 200 mg/mL. A dose of 528 mg/kg, corresponding human equivalent dose (Animal dose mg/kg = Human dose mg/kg × *K*m of human/*K*m of rat), containing 16.67 μg ferulic acid was administered orally. Where, *K*m is a correction factor obtained upon dividing body weight by surface area.

##### Collection of blood samples.

The blood samples (0.5 mL) were collected in EDTA coated tubes from tail vein (IACUC, 1999) at 0 min (pre dose), and at ¼, ½, 1, 2, 4, 6, 8, 12 and 24 h. Blood samples were centrifuged at 2500 rpm at 10 °C for 10 min to get plasma.

#### Extraction of ferulic acid from plasma

In a centrifuge tube, 500 μL plasma was mixed with 200 μL of acetonitrile by vortex for 5 s. Then, 2 mL of ethyl acetate was added and mixed by vortex for 5 s. The tube was centrifuged at 3000 rpm for 5 min at 10 °C, and the supernatant was dried with a stream of liquid nitrogen. The residue was reconstituted with 100 μL methanol.

Rat plasma (500 μL) was spiked with 1 mL of standard solutions of ferulic acid (0.4, 0.8, 1.2, 1.6 and 2.0 μg/mL) and mixed using a vortex for 5 s. Then, the analyte was extracted as that of the samples and analyzed at the aforementioned chromatographic conditions.

#### Statistical analysis

The data of enzyme activity were analyzed using one-way ANOVA with LSD multiple comparison. A *p* value less than 0.05 was considered as significantly different.

## Results

### Enzyme activity

**Figure 1.**
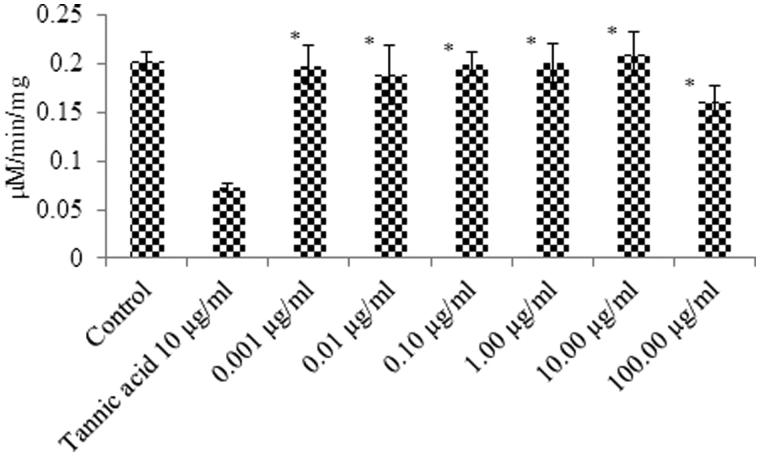
Glutathione sulfotransferase inhibition activity of control, tannic acid and different concentrations of *Kanji*, prepared from the roots of *Daucus carota* L. Each bar is a mean of three independent experiments ± SD. *(significant difference from positive control, *p* < 0.05).

The results of the GST inhibition activity of different concentrations of *Kanji,* tannic acid (positive control) and water (control) are shown in Figure 1. The activity of *Kanji* solutions (0.001–80.00 μg/mL) was not statistically different from the control, water. However, it showed a very weak activity at a concentration of 100.00 μg/mL, which was significantly lesser than tannic acid (10.00 μg/mL), a positive control (*p* < 0.05).

**Figure 2.**
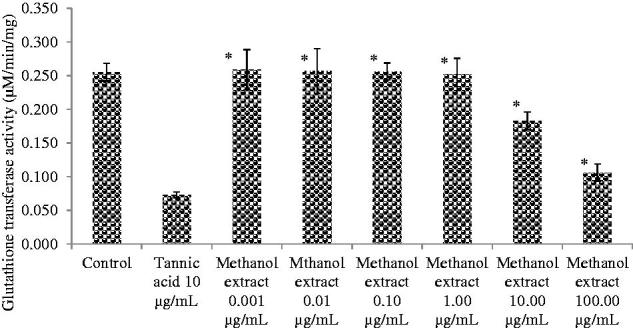
Glutathione sulfotransferase inhibition activity of control, tannic acid and different concentrations of methanol extract of the roots of *Daucus carota* L. Each bar is a mean of three independent experiments ± SD. *(significant difference from positive control, *p* < 0.05).

The results of the GST inhibition activity of methanol extract, tannic acid (positive control) and control are shown in Figure 2. The activity of the extract in lower doses (0.001–1.00 μg/mL) was comparable to that of the control (water). The extract showed enzyme inhibition activity at a concentration of 10.00 μg/mL. However, the activity of the extract at a concentration of 100.00 μg/mL was significantly lower than tannic acid (10.00 μg/mL), a positive control (*p* < 0.05).

### Identification of compound (ferulic acid)

**Figure 3.**
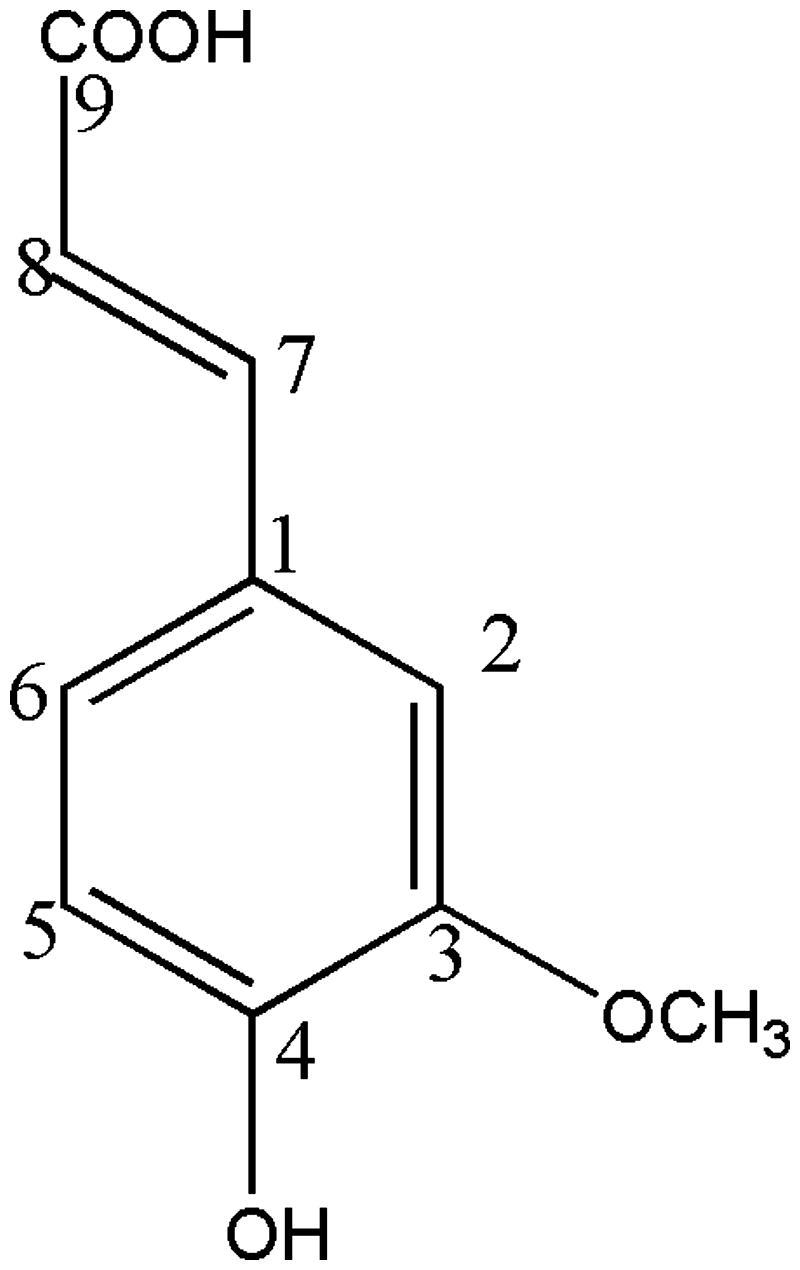
Structural formula of ferulic acid.

A single spot on TLC and a single peak in HPLC chromatogram indicated the purity of the compound. Other characteristics of the compound were as: light yellow crystalline, yield: 0.1%, MP: 168 °C, [α]D: optically inactive, no asymmetric carbon, *R*_f_: 0.79 (methanol:ethyl acetate, 1:1, v/v) and *ʎ*_max_ (methanol) nm: 218, 236, 326 nm (Supplementay 1a). FTIR (KBr) cm^−1^: 3450 cm^−1^ (–OH stretching, phenol), 1678.07 cm^−1^ (carboxylic acid C = O stretching), 1271.95 cm^−1^ (carboxylic acid C–O stretching), 1514.12 and 1425 cm^−1^ (aromatic C = C) (Supplementary 1b). ^1^H NMR (600 MHz, CD_3_OD_,_*J* in Hz), *δ*: 3.86 (s, 3H, 3 OCH_3_′), 6.19 (d, 1H, *J* = 15, 8-H′), 6.84 (d, 1H, *J* 9, 5- H), 7.01 (dd, 1H, *J* 8.3, 2, 6-H), 7.59 (d, 1H, *J* 15, 7-H′) (Supplementary 1c). ^13^C NMR (300 MHz, CD_3_OD) δ 55.88 (OCH_3_´), 109.71 (C-8), 114.89 (C-2), 115.10 (C-5′), 123.11 (C-6), 126.75 (C-1), 145.73 (C-4′), 147.06 (C-7), 148.23 (C-3), 169.57 (C-9′) (Supplementary 1d). In ^13^C NMR spectrum, the peak at 49 ppm was of the solvent. DEPT 135 (300 MHz, CD_3_OD) indicated 1 CH_3_ and 5 CH and there was no negative signal meaning the absence of CH_2_, *δ* 55.88 (OCH_3_^′^), 109.71 (C-8), 114.89 (C-2), 115.10 (C-5^′^), 123.12 (C-6), 145.78 (C-4′) (Supplementary 1e). DEPT 90 (300 MHz, CD_3_OD) indicated 5 CH, 109.71 (C-8), 114.89 (C-2), 115.10 (C-5´), 123.12 (C-6), 145.78 (C-4′) (Supplementary 1f). MS (ESI full scan): *m/z* (100%) = 194 [M^+^] (100), 179 (21), 161 (7), 133 (32), 105 (14), 89 (15). GC-MS: Relative retention 19.4 min. HRMS-FAB: *m/z* 194 [M^+^, base peak] calcd. for C_10_H_10_O_4_ (Supplementary 1g). This data indicated that the isolated compound was ferulic acid (Sajjadi et al. 2012). The structure of ferulic acid is shown in Figure 3.

### Ferulic acid contents of *Kanji,* extracts and fractions of plant roots

Two types of columns and different types of mobile phases were attempted to improve resolution (*R*s), retention time (*t*_R_), peak shape and symmetry. The mobile phase (water, methanol and phosphoric acid, 300:200:2.5, v/v/v), column and chromatographic conditions used in the present study produced optimum results. The detection was carried out using DAD at 220, 260 and 320 nm and fluorescent light detector (FLD); operated at an excitation wavelength of 250 nm and emission wavelength of 410 nm. Both of the detectors gave appropriate response, though the response of the FLD was comparatively better. The method was found to be linear in the whole range investigated with correlation coefficient from 0.9978–0.998. LOD and LOQ values of ferulic acid determined using statistical methods were 0.43 and 1.30 μg/mL, respectively. The recovery was 98.52–100.00% with relative standard deviation less than 5%. Intra- and inter-day accuracy was 98.20–100.72% (RSD <5%) and 98.47–101.27% (RSD <5%), respectively. These results indicate that the method is specific, sensitive, repeatable and reproducible.

Contents of ferulic acid determined in different types of *Kanji* samples, extracts and fractions of the roots of the plant are presented in Table 1. Lab-made *Kanji* contained higher contents of ferulic acid as compared to homemade and commercial *Kanji* samples. This difference may be due to the use of different quantities of water used in preparing the beverage. On the other hand, methanol extracts obtained by sequential extraction contained higher contents of ferulic acid. Hexane and chloroform extracts were not having any quantifiable amount of ferulic acid. Among the fractions, the ferulic acid was higher in butanol and water fractions as compared to other fractions.

**Table 1.** Contents of ferulic acid in *Kanji* and extracts and fractions of roots of *Daucus carota* L. samples (*n* = 3).

Sample	mg/g	SD
*Kanji* (Lab-made)	0.316	0.03
*Kanji* (Homemade)	0.291	0.04
*Kanji* (Com-1)	0.237	0.035
*Kanji* (Com-2)	0.227	0.028
*Kanji* (Com-3)	0.241	0.023
*Kanji* (Com-4)	0.222	0.029
*Kanji* (Com-5)	0.217	0.041
*Kanji* (Com-6)	0.222	0.043
Hexane extract	0	0
Chloroform extract	0	0
Methanol extract	0.770	0.034
Hexane fraction	0.133	0.023
Chloroform fraction	0.167	0.029
Ethyl acetate fraction	0.128	0.04
*n*-butanol fraction	0.222	0.038
Water fraction	0.193	0.053

Com (Commercial).

The comparison of the enzyme inhibition activity of *Kanji* and methanol extract indicated a correlation of the activity with the contents of ferulic acid. The contents of ferulic acid were significantly higher in methanol extract as compared to *Kanji*; hence higher enzyme inhibition. The *Kanji* samples used in the present study were found to have solid contents 14–36 mg/mL. Lab-made *Kanji* having mass/mL of 36 mg/mL contained 40 μg/mL ferulic acid. The concentration of *Kanji* (80 μg/mL), which did not show enzyme inhibition, contained 0.0025 μg ferulic acid. So, if ferulic acid concentration reaches above this concentration in the blood may inhibit the GST, which needs to be confirmed by bioavailability studies.

### Oral bioavailability of ferulic acid from *Kanji*

**Figure 4.**
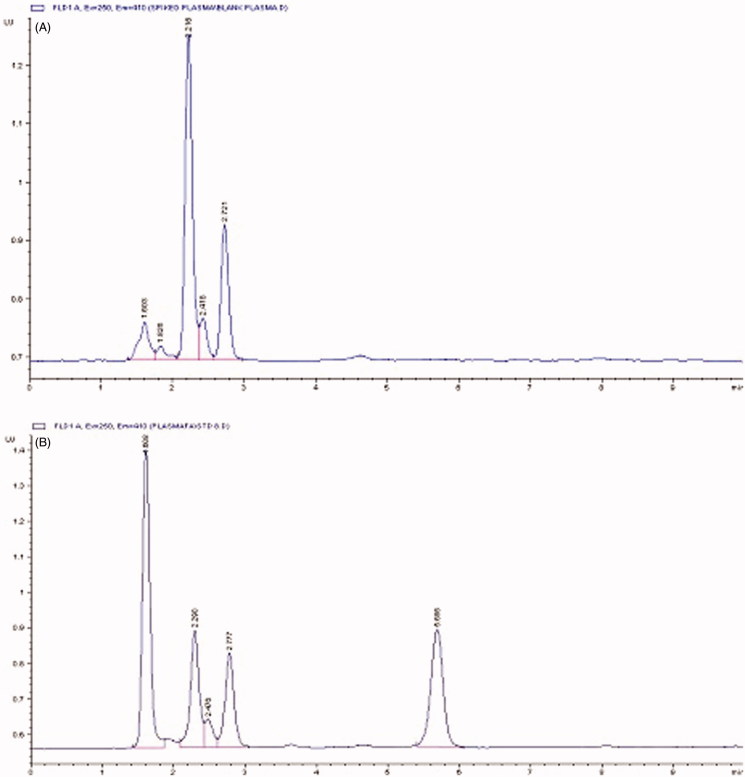
HPLC chromatograms of (a) blank plasma and (b) ferulic acid-spiked plasma.

A combined approach, deproteination and liquid–liquid extraction, was applied for extraction of ferulic acid from plasma. After deproteinization with acetonitrile, extraction was achieved by ethyl acetate, which was removed and the residue was reconstituted with methanol before analysis. The method of extraction proved to be appropriate because no interference of endogenous compounds of the plasma was noticed on the peak of ferulic acid. The chromatograms of blank plasma and ferulic acid extracted from the plasma are given in Figure 4.

The calibration curve used for the determination of ferulic acid from plasma gave the linear regression equation, *Y* = 10.075 × −2.25, and correlation coefficient *R*^2 ^=^ ^0.9983. The mean per cent recovery was 95.52–97.5% with relative standard deviation less than 5%. The intraday and inter–day accuracy was 97.66–100.13 and 97.18–100%, respectively, with relative standard deviation less than 5%. These results indicate that the method is reliable, repeatable and reproducible.

In the present study, no peak for ferulic acid was observable on chromatogram corresponding the retention time of ferulic acid. It was only detected in samples, collected after 30 min but the concentration was not quantifiable reliably. This indicated that the concentration of ferulic acid in blood could not reach to the measurable level during entire time interval from 0.50 to 24 h. This finding was in line with a study where the ferulic acid from diet could not be detected when given in lower doses (10.0 μmol/dL) (Adam et al. 2002). Contrary to this, ferulic acid is reported to be readily absorbed (Zhao et al. 2004) and this absorption is from stomach, jejunum and ileum (Spencer et al. 1999; Zhao et al. 2004). But, the difference was that these studies were carried out on the ferulic acid *per se*, using *in situ* or *ex vivo* absorption models, and not from the herbal extracts.

## Discussion

The results of the present study showed that *Kanji* at a concentration of 0.001–80.00 μg/mL and methanol extract at a concentration of 0.001–10.00 μg/mL did not inhibit the activity of GST. *Kanji* (80 μg) contains 0.0025 μg of ferulic acid, and up to this level it is not expected to have an effect on the enzyme. However, *Kanji* having ferulic acid in higher quantity can inhibit the activity of GST. Therefore, ferulic acid contents may be used as a guide to prepare standardized beverage, which is safer to be used with other co-administered drugs, substrates of the enzyme. The results of the present study may be correlated to that of *in vivo* studies, because freshly isolated hepatocytes have all the co-factors and sub-families of cytochrome P_450s_. It was further supported by the findings of a previous study, which indicated that cytochrome P_450s_ enzymes could perform activity during *in vitro* experiments as in the living system (Kato et al. 2005).

Methanol extract showed higher enzyme inhibition activity as compared to *Kanji,* which is an aqueous-based preparation of roots of the plant. This may be due to the extraction of nonpolar compounds with petroleum ether and chloroform leaving polar components including ferulic acid in methanol extract. This was supported from the results of the present study because ferulic acid was not detected in petroleum ether and chloroform extracts. These findings suggest that aqueous extract of roots of the plant may be dried and used whenever required for the preparation of *Kanji* round the year, however, it needs further investigations to address the stability issues of aqueous extracts. The correlation between the enzyme inhibition activity and ferulic acid contents indicates that this compound may be used to produce standardized *Kanji*.

The HPLC method used for the quantification of ferulic acid was simple, hence could be used to assess the production of *Kanji* with controlled ferulic acid contents. The analysis proved that *Kanji* samples vary in terms of ferulic acid contents. This variation was due to two main reasons; the use of different quantities of carrots and the dilution made at the time of selling or addition of ice in *Kanji* to prevent its fermentation. By maintaining ferulic acid contents, this beverage may get in the main stream of evidence-based complementary medicines. The higher level of ferulic acid in *Kanji* and in the blood may inhibit GST activity. To determine the levels of ferulic acid in the blood from the oral route, bioavailability studies were performed, but we could not find its measurable quantities in the blood.

The literature review indicated a number of facts regarding ferulic acid contents in the blood. Zhao and Moghadasian (2008) have reported that ferulic acid is rapidly metabolized majorly to ferulic acid-glucuronides and sulphates (Rondini et al. 2002; Zhao et al. 2003), and circulate in blood only as conjugated forms (Adam et al. 2002). An efficient absorption of ferulic acid (Zhao et al. 2004) coupled with a fast distribution, i.e., within 30 min (Chang et al. 1993) may lead to its fast metabolism (conjugation) thus a very low bioavailability was found (Chang et al. 1993; Zhao et al. 2004; Li et al. 2011). This was further supported by its short half-life of 10–30 min depending on the route of administration (Chang et al. 1993; Zhao et al. 2003). The conjugated- or sulphonated-ferulic acid in plasma could not be detected as the intact compound. Literature did not support the enzymatic degradation of the ferulic acid which could be regarded as another possible reason for lack of its presence in the blood (Adam et al. 2002). The acidic pH-led decomposition of ferulic acid was not supported by its p*Ka* value. The p*K* of the ferulic acid (p*Ka* 4) indicates that it remains un-disassociated at low gastric pH which may favour its absorption by passive diffusion (Tsuji & Tamai 1996). Catabolism of a moiety by microflora of the small intestine could be another reason for the lack of its appearance in the blood (Adam et al. 2002). Chesson et al. (1999) reported that the intestinal microflora catabolize ferulic acid. However, lack of its appearance in blood could not be ascribed to microfloral catabolism, since it was efficiently absorbed from the stomach (Zhao et al. 2004). Moreover, owing to a p*Ka* 4, ferulic acid does not sustain its un-dissociated form in the small intestinal (pH >5), hence cannot be absorbed through passive diffusion (Tsuji & Tamai 1996; Zhao & Moghadasian 2008). Van der-Logt et al. (2003) reported that lower bioavailability of the dietary ferulic acid had led to imply the binding of ferulic acid with the unknown compounds that were possibly present in *Kanji* could be a reason for the lack of its appearance in the blood. The excretion of ferulic acid mainly through urine, Rondini et al. (2002) and Zhao et al. (2004), supported to find cumulative urinary excretion to estimate the bioavailability of dietary ferulic acid, which might be a suggestion for the future study. Keeping all the above in view, the most possible reason for the lack of manifestation of ferulic acid in blood was its low contents in *Kanji*.

## Conclusion

It is concluded from the results of the present study that *Kanji* does not inhibit the glutathione sulphotransferase activity. Moreover, ferulic acid may be used as a correlative analytical standard to control its contents to avoid GST inhibition activity. Poor oral bioavailability of ferulic acid after administering *Kanji* is due to its low contents in the administered dose.

## Supplementary Material

Khalid_Hussain_et_al_supplemental_content.zip
