# Supplementary material for: Glutathione sulfotransferase inhibition activity of a self-fermented beverage, Kanji
Source: Pharm Biol. 2016 Dec 12;55(1):547–53. doi: 10.1080/13880209.2016.1257030 (PMC6130599; doi:10.1080/13880209.2016.1257030)
Supplement: Khalid_Hussain_et_al_supplemental_content.zip [file IPHB_A_1257030_SM1863.zip › Khalid Hussain et al supplemental content.pdf]

Supporting Information

Glutathione sulfotransferase inhibition activity and characterization of *Kanji* and extracts of roots of *Daucus carota* L.

Abida Latif, Khalid Hussain*, Naureen Shehzadi, Muhammad Tanveer Khan, Muhammad Islam, Nadeem Irfan Bukhari, Hamid Saeed, Rukhsana Anwar

University College of Pharmacy, University of the Punjab, Allama Iqbal Campus, Lahore-54000, Pakistan

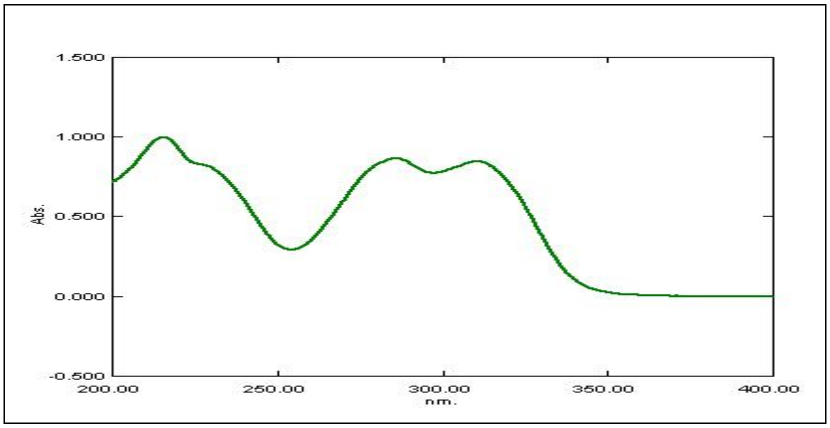

Supplementary 1a UV scan of ferulic acid isolated from methanol extract of roots of *Daucus carota* L.

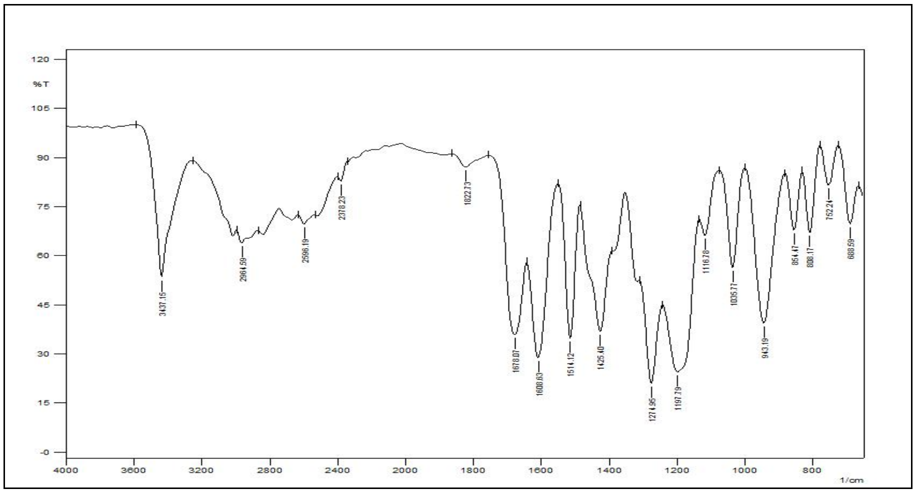

Supplementary 1b FTIR spectrum of ferulic acid isolated from methanol extract of roots of *Daucus carota* L.

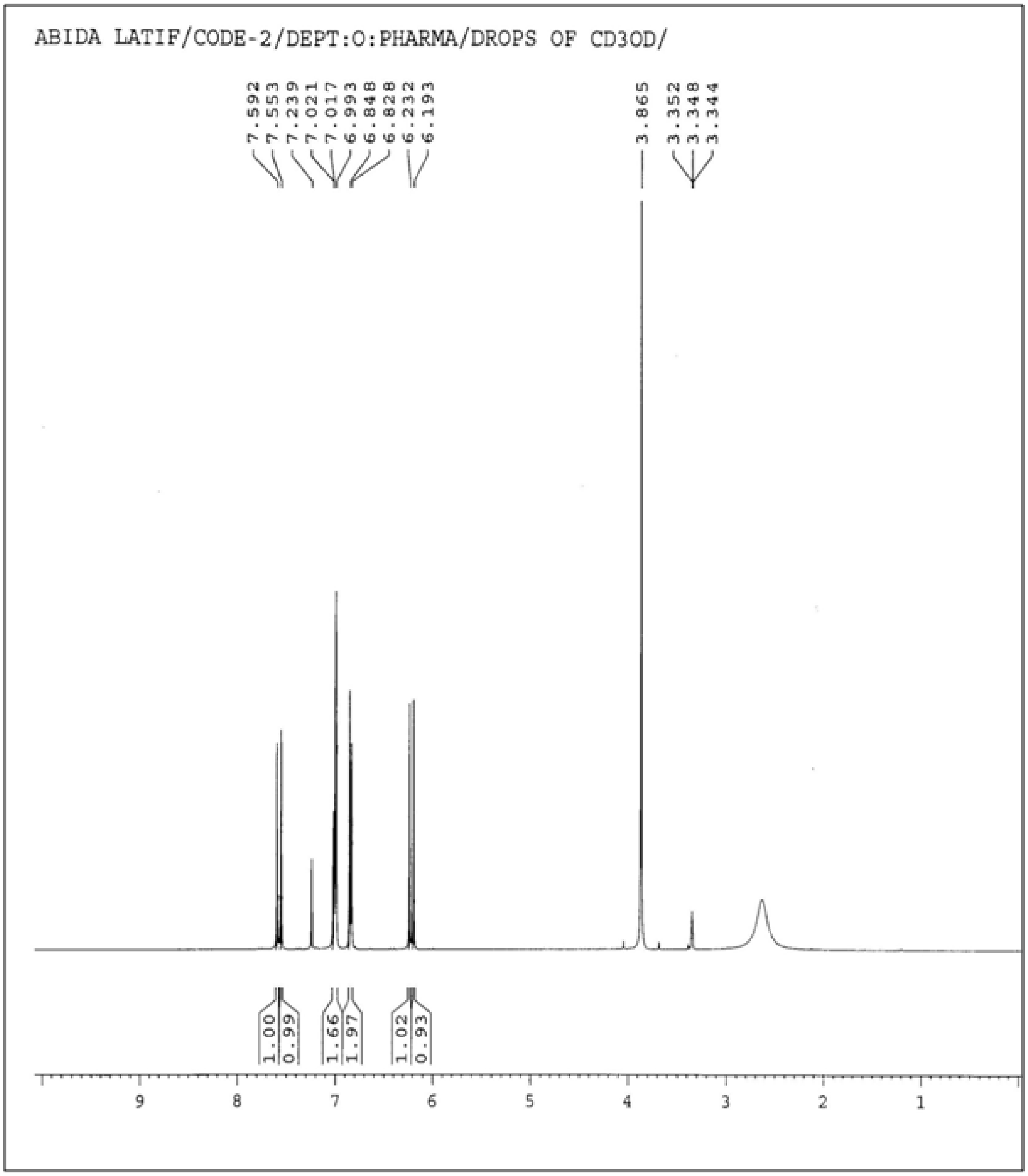

Supplementary 1c ^{1}H NMR spectrum of ferulic acid isolated from methanol extract of roots of *Daucus carota* L.

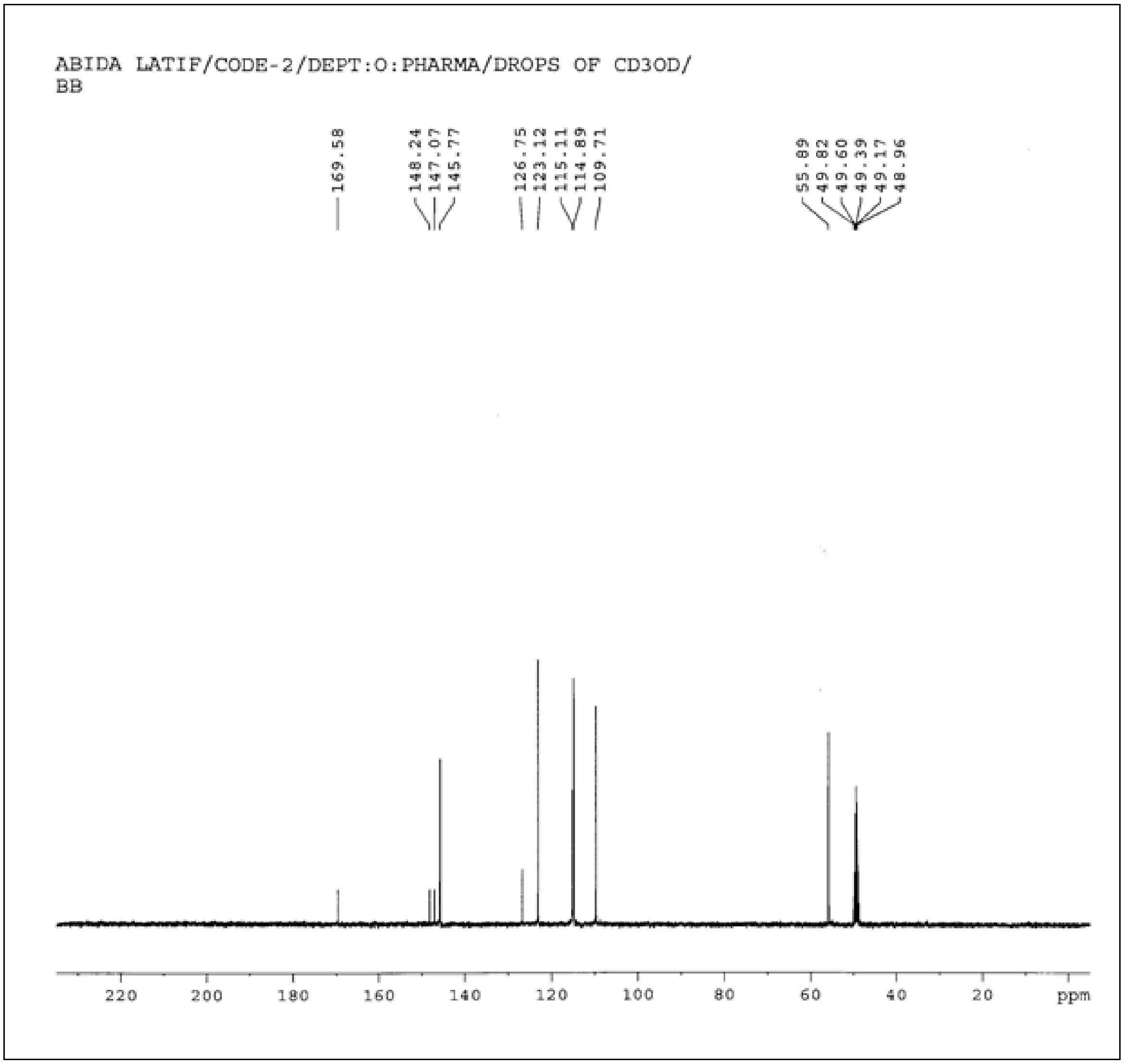

Supplementary 1d ^{13}C NMR spectrum of ferulic acid isolated from methanol extract of roots of *Daucus carota* L.

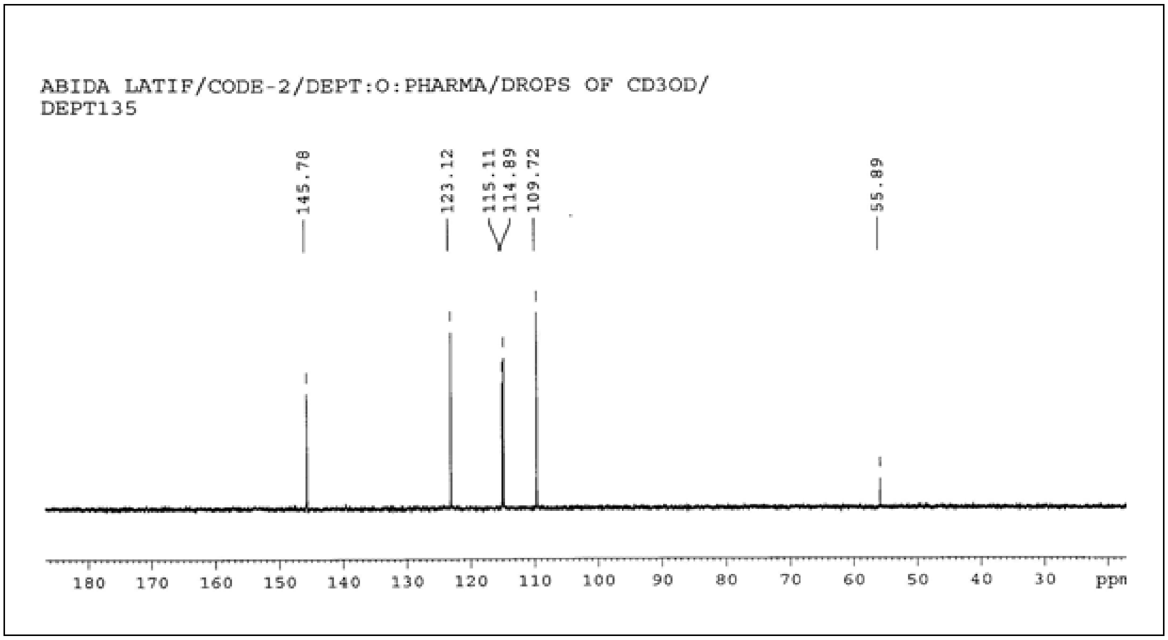

Supplementary 1e ^{13}C DEPT 135 spectrum of ferulic acid isolated from methanol extract of roots of *Daucus carota* L.

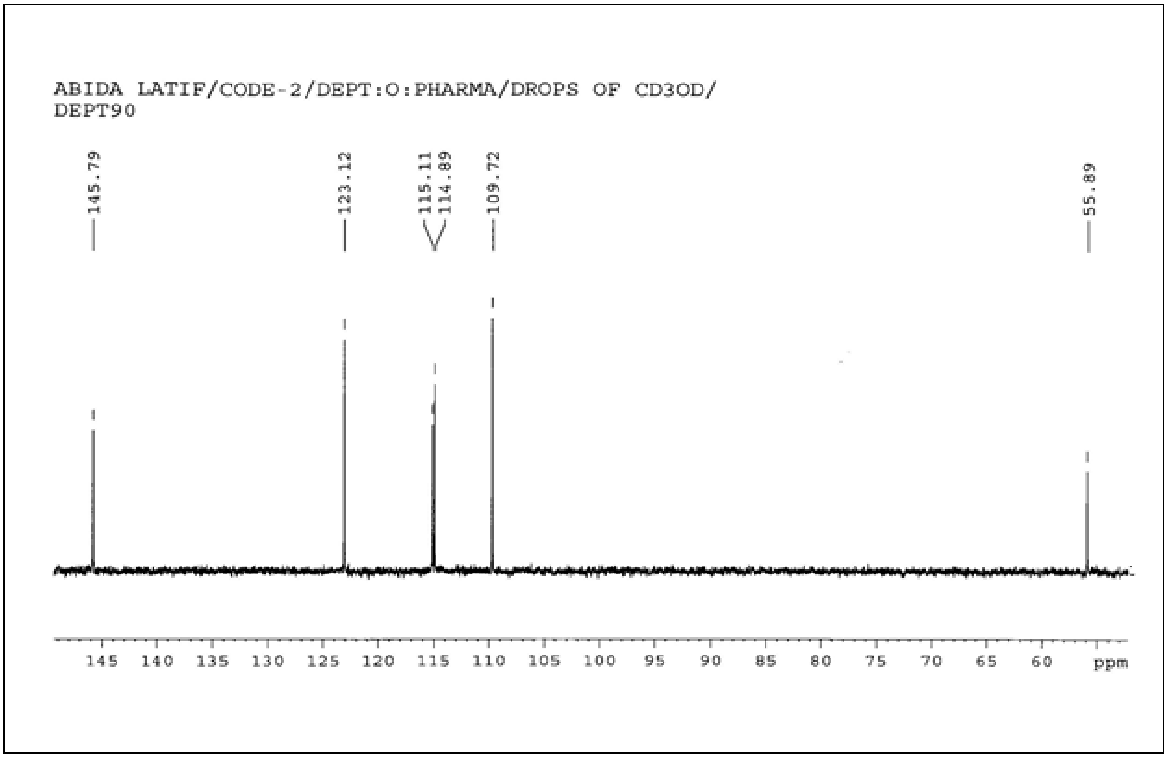

Supplementary 1f ^{13}C DEPT 90 spectrum of ferulic acid isolated from methanol extract of roots of *Daucus carota* L.

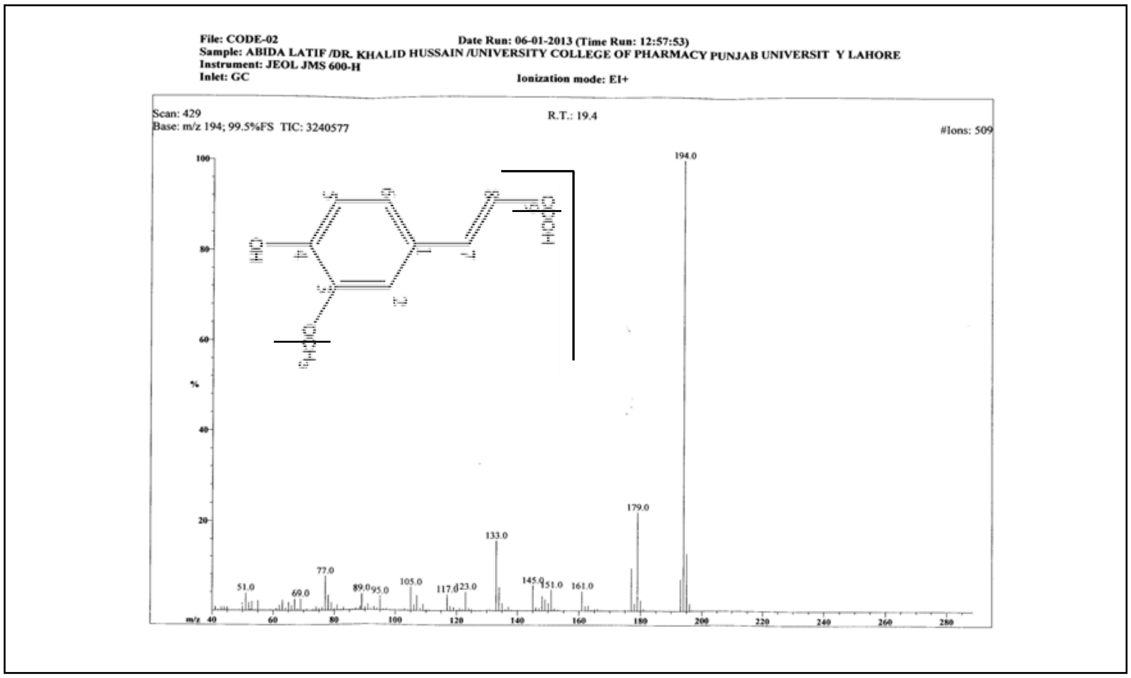

Supplementary 1g GC/MS ionogram of ferulic acid isolated from methanol extract of roots of *Daucus carota* L.
